# Compression fracture of the T12 vertebra combined with diaphragmatic hernia after low-energy trauma: a case report and literature review

**DOI:** 10.3389/fsurg.2026.1851167

**Published:** 2026-08-12

**Authors:** Zhenghui Li, Rugang Fu, Wei Huang, Zhigang Cheng

**Affiliations:** 1 Guizhou University of Traditional Chinese Medicine, Guiyang, China; 2 Department of Orthopedics, Anyue County Hospital of Traditional Chinese Medicine, Ziyang, China; 3 Department of Orthopedics, The Second Affiliated Hospital, Guizhou University of Traditional Chinese Medicine, Guiyang, China

**Keywords:** diaphragmatic hernia, minimally invasive surgery, missed diagnosis, percutaneous kyphoplasty, thoracolumbar vertebral fracture, thoracoscopy

## Abstract

**Background:**

Vertebral compression fractures are common in middle-aged and elderly patients with osteopenia; however, their coexistence with diaphragmatic hernia (DH) is rare and easily overlooked, especially when diaphragmatic hernia presents without typical respiratory or gastrointestinal symptoms.

**Case presentation:**

We report a case of a 56-year-old woman with osteopenia who developed a T12 vertebral compression fracture combined with acute exacerbation of a pre-existing diaphragmatic defect following a ground-level fall. The patient presented with persistent thoracolumbar pain and right lower limb pain for five days, with no classic symptoms of diaphragmatic hernia on admission. The key diagnostic clue was identified on initial chest radiography, which demonstrated reduced left lung volume and elevation of the left hemidiaphragm; subsequent chest computed tomography (CT) confirmed the diagnosis, revealing a left diaphragmatic defect with herniation of the greater omentum and colon into the thoracic cavity. The patient first underwent video-assisted thoracoscopic surgery (VATS) for diaphragmatic hernia repair, followed by percutaneous kyphoplasty (PKP) for the vertebral fracture after her condition stabilized. She recovered well and was discharged after systematic treatment.

**Conclusion:**

This case indicates that for patients with thoracolumbar vertebral fractures caused by blunt trauma, chest CT should be performed promptly when abnormal findings are detected on chest radiography or when patients present with nonspecific abdominal or thoracic symptoms, to exclude concomitant diaphragmatic hernia, achieve early diagnosis, and reduce the risk of diagnostic delay.

## Introduction

Vertebral compression fractures are prevalent skeletal disorders in middle-aged and elderly individuals with reduced bone mineral density, with a particularly high incidence in postmenopausal women (1, 2). These fractures typically result from minor or low-energy trauma and manifest as acute thoracolumbar pain, which may radiate to the thoracic and abdominal wall and mimic symptoms of intrathoracic or intra-abdominal diseases (3).

Diaphragmatic hernia is a rare but potentially life-threatening complication of thoracoabdominal trauma. Epidemiological data show diaphragmatic rupture complicates 0.8%–5% of all thoracoabdominal injuries, and nearly one-third of such injuries evolve into chronic delayed diaphragmatic hernia with insidious onset (4, 5). Blunt trauma including high falls, motor vehicle collisions, and direct torso impact accounts for the vast majority of traumatic diaphragmatic defects. The concurrent presentation of thoracolumbar vertebral fracture and diaphragmatic hernia is exceptionally rare, and diagnosis is clinically challenging: pain and limited motion caused by spinal injury often mask the subtle and atypical signs of diaphragmatic hernia, which may lead to delayed recognition (6, 7).

Most previously reported cases of combined spinal fracture and diaphragmatic hernia occurred after high-energy trauma, and data on cases caused by low-energy falls remain scarce. In this report, we describe the diagnostic and therapeutic course of a patient with T12 vertebral compression fracture and diaphragmatic hernia after a ground-level fall, and conduct a brief literature review to highlight the clinical characteristics of this rare combined injury and provide reference for clinical practice.

## Case presentation

### Patient information

A 56-year-old female patient presented to the emergency department on July 18, 2023, with persistent thoracolumbar pain accompanied by right lower limb pain for five days after a ground-level fall. She reported continuous dull thoracolumbar pain, aggravated by movement, occasional abdominal discomfort, constipation lasting four days, and normal urination. Initial lumbar x-rays suggested a T12 vertebral fracture. Her medical history included surgery for an ectopic pregnancy 21 years earlier at the Third People's Hospital of Guizhou Province (details unknown), with uneventful recovery. Physical examination revealed mild thoracolumbar kyphosis and positive tenderness and percussion pain in the thoracolumbar region, especially at the T12 vertebra. Palpation elicited tenderness in the region extending from the T10 to L5 spinous processes and bilateral paraspinal areas (1.5 cm from the midline). Lumbar movement was limited. The straight leg raise test was negative bilaterally. Local tenderness was noted in the right lower limb and knee, and the patellar grinding test was positive. Laboratory tests showed normal urinalysis and renal function. Urinary system ultrasound revealed no abnormalities in the kidneys, ureters, or bladder. Lumbar x-rays indicated lumbar degeneration and vertebral flattening at T12 and L1, and further MRI examination was recommended. The preliminary admission diagnosis was a T12 vertebral compression fracture. The detailed clinical course of the patient is summarized in Table 1.

**Table 1:** Clinical timeline of the patient.

Time point	Key events
Admission day (Day 0)	Presented to the emergency department with thoracolumbar pain after a ground-level fall and was initially admitted to the spine surgery team. Lumbar plain radiography suggested a T12 vertebral fracture, while routine chest radiography revealed an elevated left hemidiaphragm with reduced left lung volume (Figure 1). Given this abnormal finding, chest CT was scheduled for the following day.
Hospital day 1 (Day 1)	Chest CT was performed, which confirmed a left diaphragmatic defect with herniation of omental and colonic tissues (Figure 2). Thoracolumbar MRI also confirmed acute T12 compression fracture with bone marrow edema (Figure 3). Multidisciplinary consultation between spine and thoracic surgery determined that the diaphragmatic hernia carried higher immediate risk.
Hospital day 2 (Day 2)	The patient was transferred to the thoracic surgery team.Underwent video-assisted thoracoscopic surgery (VATS) for diaphragmatic hernia repair.
Postoperative day 10	Transferred back to the spine surgery department for further management of the vertebral fracture. During the thoracic recovery period, the patient received bed rest and analgesic treatment.
Postoperative day 11	Underwent percutaneous kyphoplasty (PKP) for the T12 vertebral compression fracture.
Postoperative day 18	Bone mineral density test showed osteopenia (T-score: –1.8); oral calcium carbonate with vitamin D3 supplementation was initiated.
Postoperative day 32	Patient recovered satisfactorily and was discharged; total hospital stay: 34 days.
3-month follow-up	Patient had resumed daily household activities; no recurrence of hernia or persistent back pain.

## Clinical timeline

### Diagnostic assessment

#### Physical examination and initial laboratory findings

On admission, physical examination revealed thoracolumbar kyphosis, with tenderness and percussion pain over the T10–L5 spinous processes and bilateral paraspinal areas (1.5 cm from the midline), most pronounced at T12. Lumbar spinal motion was limited. The straight leg raise test was negative bilaterally. Local tenderness was noted in the right lower limb and knee, and the patellar grinding test was positive. Laboratory tests, including complete blood count, coagulation profile, and liver and kidney function tests, were all within normal limits. Urinalysis and urinary ultrasound showed no abnormalities, ruling out infection, coagulopathy, and urological injuries.

#### Imaging studies and Key findings

Lumbar plain radiographs obtained on admission revealed lumbar degeneration and flattening of the T12 and L1 vertebrae, with approximately 32.4% loss of T12 vertebral body height, initially suggesting a T12 compression fracture.

**Figure 1:**
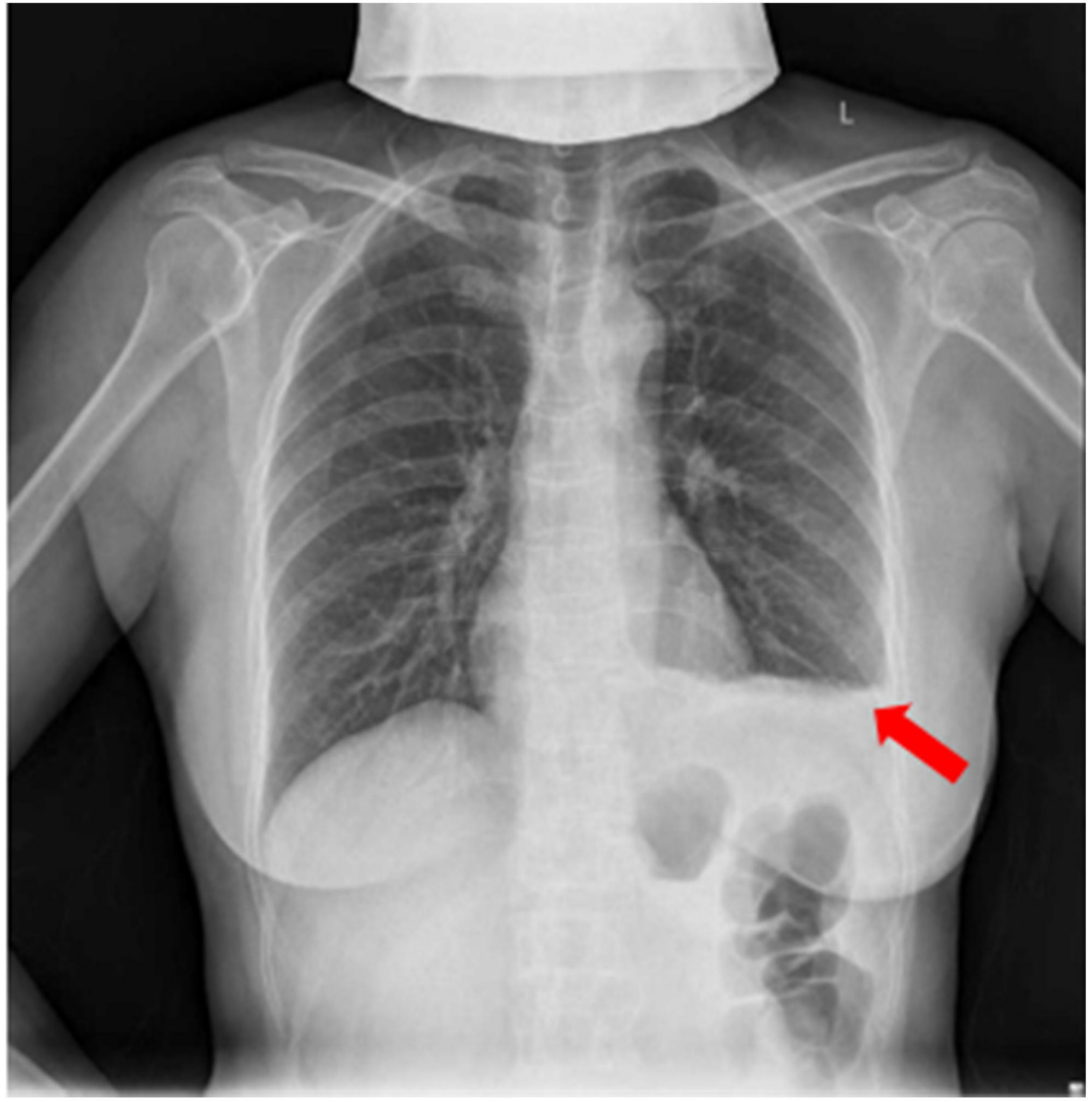
Chest radiography on admission showing reduced left lung volume and elevation of the left hemidiaphragm.

Chest radiography performed routinely on admission demonstrated reduced left lung volume and marked elevation of the left hemidiaphragm (Figure 1). This finding could not be explained by the vertebral fracture alone and raised suspicion of diaphragmatic or intrathoracic pathology, prompting further evaluation.

**Figure 2:**
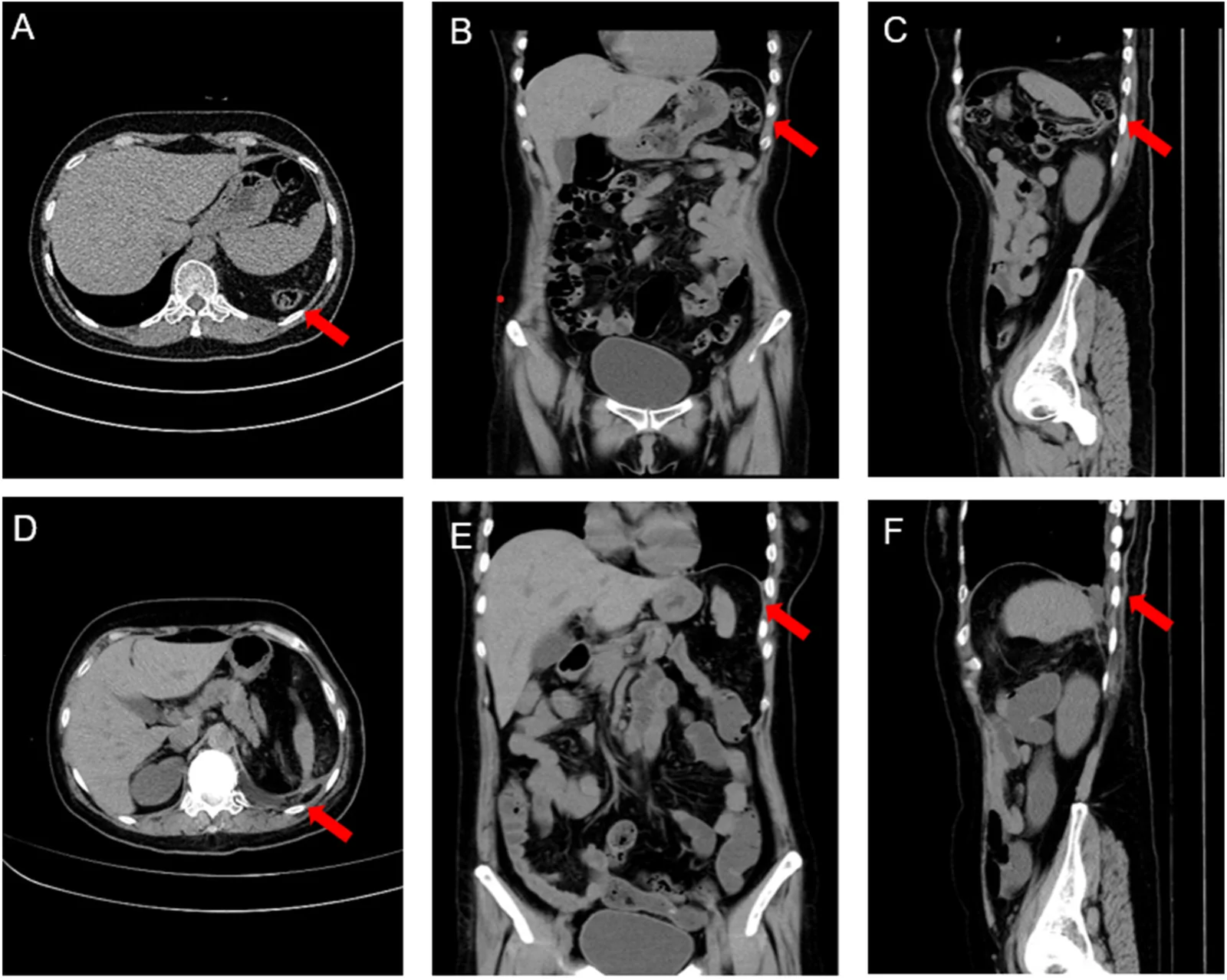
Preoperative and postoperative chest CT images. **(A–C)** Preoperative sagittal, coronal, and axial views demonstrating left diaphragmatic hernia with herniation of the colon and greater omentum. **(D–F)** Postoperative sagittal, coronal, and axial views showing complete repair of the left diaphragmatic defect.

Chest computed tomography (CT) obtained on hospital day 1 clearly showed a left diaphragmatic defect with interruption of diaphragmatic continuity, and herniation of the greater omentum and part of the colon into the left thoracic cavity, confirming the diagnosis of left diaphragmatic hernia (Figures 2A–C).

**Figure 3:**
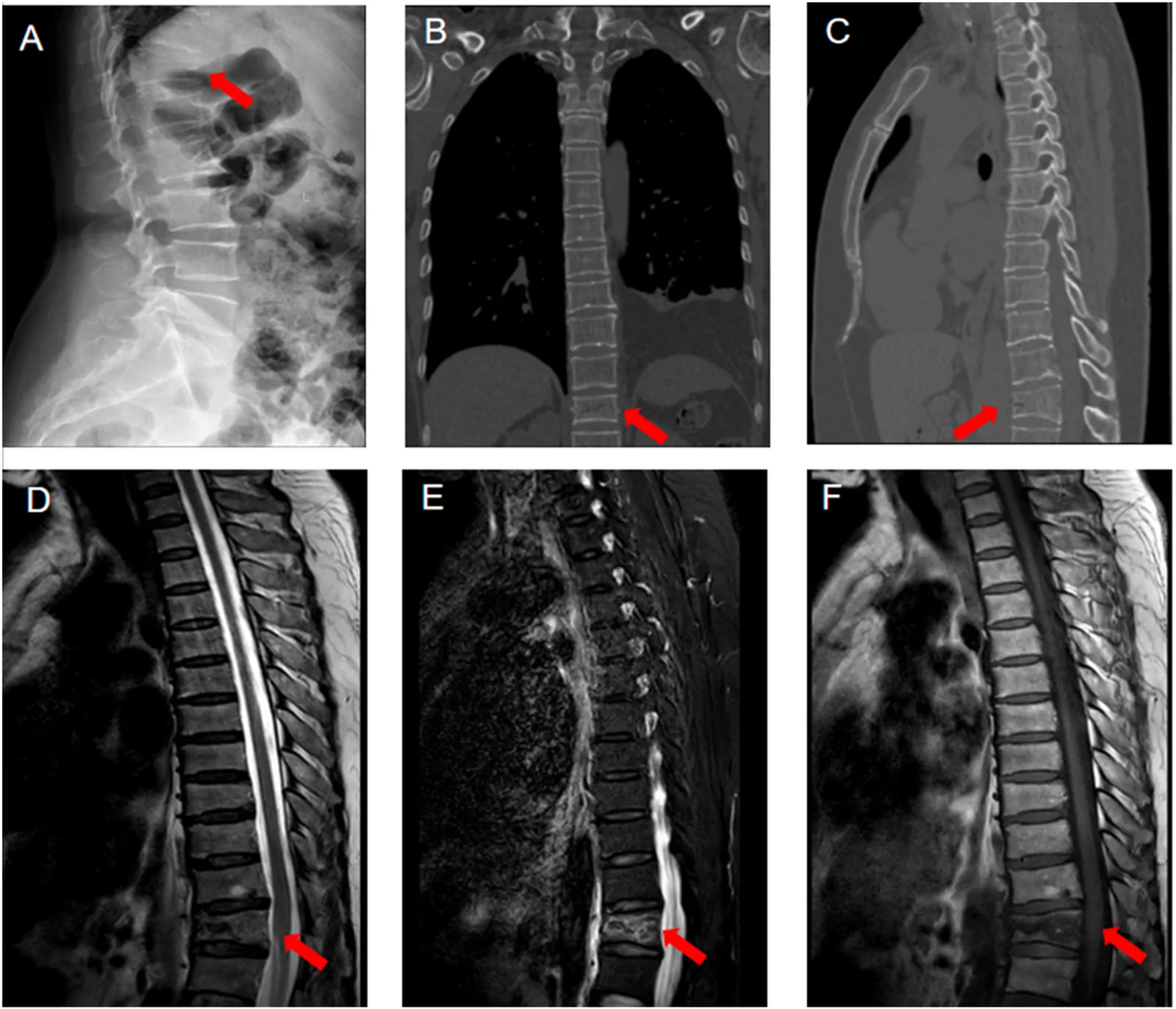
Plain radiography, CT, and MRI images **(A–F)** demonstrating T12 vertebral compression fracture with acute bone marrow edema.

Thoracolumbar magnetic resonance imaging (MRI) performed on hospital day 1 confirmed an acute T12 compression fracture, with bone marrow edema. The posterior vertebral wall was intact, and there was no spinal canal compromise or neurological involvement (Figures 3D–F).

Abdominal ultrasound obtained on admission showed no abnormalities in the kidneys, ureters, or bladder, excluding urogenital causes of abdominal pain.

#### Diagnostic reasoning

The patient sustained a lowenergy groundlevel fall that directly caused a T12 vertebral compression fracture. An elevated hemidiaphragm visualized on chest radiograph raised strong clinical suspicion of diaphragmatic injury, which was subsequently confirmed as a left diaphragmatic hernia via computed tomography. Given the lowenergy injury mechanism, the hernia was deemed to represent an acute exacerbation of preexisting occult diaphragmatic weakness, rather than a *de novo* traumatic tear secondary to highenergy trauma.

#### Differential diagnosis

Acute abdomen (e.g., intestinal obstruction or perforation): The patient had no severe abdominal pain, guarding, nausea, vomiting, or fever, and CT showed no free air or bowel ischemia, thus ruling out these conditions.

Diaphragmatic paralysis: Although it can cause hemidiaphragm elevation, CT confirmed an anatomical defect with visceral herniation, excluding this diagnosis.

#### Diagnostic challenges

The diagnostic challenges in this case were threefold: (1) the patient presented with no classic symptoms of diaphragmatic hernia, such as respiratory distress, nausea, vomiting, or bowel obstruction; (2) the severe pain from the vertebral fracture masked the mild abdominal complaints; and (3) the chest radiograph showed only a non-specific elevation of the hemidiaphragm, which could easily be overlooked. However, prompt recognition of the chest radiographic abnormality and timely CT examination enabled the correct diagnosis of the combined injury.

### Therapeutic intervention

#### Multidisciplinary treatment decision

After consultation between the spine surgery and thoracic surgery teams, the treatment priority and strategy were determined as follows: Specifically, the following clinical and radiological assessments informed this decision.

Regarding the diaphragmatic hernia: although the patient presented without respiratory compromise, bowel obstruction, incarceration, torsion, necrosis, or progressive lung compression at admission, and the herniated colon was not obstructed and showed no signs of perforation on imaging, the narrow hernial ring created a potential risk of incarceration, torsion, or necrosis of the herniated viscera, warranting timely repair (18, 19).

Regarding the spinal injury: the T12 compression fracture was mechanically stable; MRI demonstrated no posterior ligamentous complex (PLC) injury, no spinal canal compromise, and no neurological compression, confirming that definitive spinal treatment could be safely deferred. During thoracic treatment, strict bed rest and optimized analgesic therapy were maintained, and no spinal precautions or thoracolumbar brace was applied, given the stability of the fracture and poor tolerance of bracing in the early postoperative thoracic period (21, 22).

#### Stage 1: VATS diaphragmatic hernia repair (hospital day 2)

Under general anesthesia, the patient was placed in the right semi-lateral position. Three incisions were made: a 1.5-cm observation port at the 7th intercostal space along the mid-axillary line, a 2-cm operating port at the 4th intercostal space along the anterior axillary line, and a 2-cm auxiliary operating port at the 8th intercostal space along the posterior axillary line.

Thoracoscopic exploration showed incomplete expansion of the left lower lung. The greater omentum and colon had herniated into the left thoracic cavity through a defect located at the junction of the left diaphragm and the thoracic aorta. Local fibrous adhesions were observed between the herniated contents and the margin of the diaphragmatic defect. Due to the narrow hernial ring and tight adhesions, direct reduction carried a high risk of intestinal rupture and bleeding. Therefore, the auxiliary incision at the 8th intercostal space was extended to approximately 8 cm. Under direct vision, adhesiolysis was performed with electrocautery, and the hernial ring was widened. The herniated greater omentum and colon were completely reduced into the abdominal cavity.

The diaphragmatic defect was repaired with interrupted non-absorbable sutures, and the margin of the defect was fixed to the posterior chest wall to achieve complete closure of the defect and separation of the thoracic and abdominal cavities. No prosthetic mesh was used.

After thorough hemostasis with absorbable oxidized cellulose and 150 mL of biological hemostatic sealant, a single chest drainage tube was placed through the 7th intercostal observation port. The extended incision was closed with absorbable sutures in the muscular layer to reduce postoperative tension, and remaining incisions were closed layer by layer. Total estimated intraoperative blood loss was approximately 50 mL, with no blood transfusion required and no intraoperative complications. The procedure was completed uneventfully.

#### Rationale for thoracoscopic surgical approach

VATS was selected over an abdominal approach based on two core clinical considerations. First, the defect was located at the posteromedial left diaphragm adjacent to the thoracic aorta; the transthoracic approach provided unobstructed visualization of the entire defect margin and facilitated precise, tension-free primary repair under direct vision. Second, the patient exhibited no clinical or radiological signs of intra-abdominal organ injury or peritonitis, rendering formal abdominal exploration unnecessary (5, 23, 24).

#### Stage 2: percutaneous kyphoplasty (postoperative day 11)

Under local infiltration anesthesia, the patient was placed in the prone position with chest and abdominal pads. Under C-arm fluoroscopic guidance, bilateral transpedicular punctures were performed, with needle tips positioned at the posterior one-third of the T12 vertebral body.

After sequential dilation, balloon inflation was performed in the anterior one-third of the vertebral body. Intraoperative dynamic fluoroscopy showed satisfactory balloon expansion without contrast leakage. Bone cement was injected at the “pull-wire stage”, with 4.0 mL injected via the left pedicle and 4.2 mL via the right pedicle. Satisfactory cement distribution was observed, with no cement leakage into the spinal canal or paravertebral veins.

**Figure 4:**
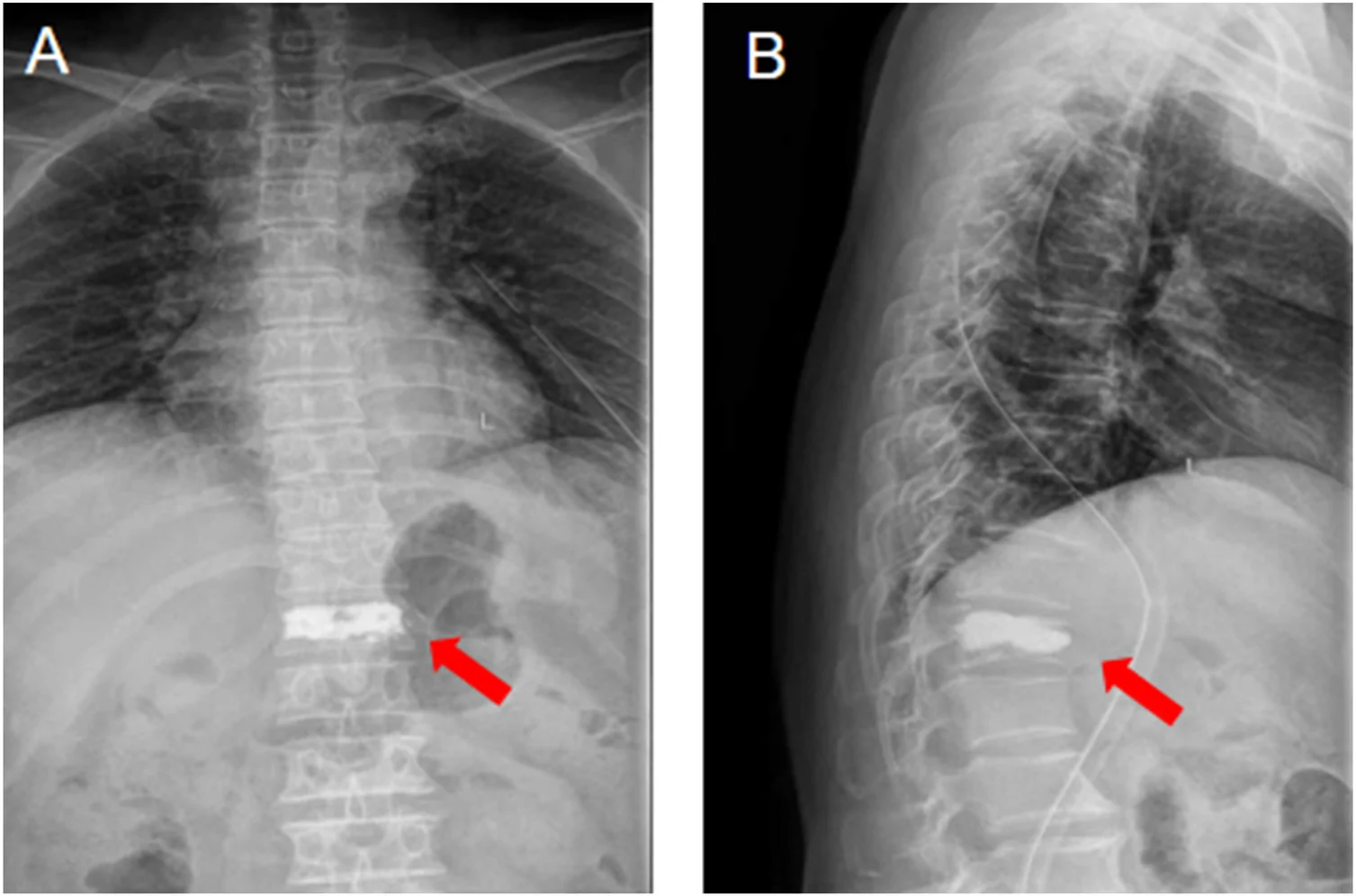
Postoperative anteroposterior **(A)** and lateral **(B)** thoracolumbar plain radiographs after PKP, showing satisfactory cement distribution and restored vertebral height.

The patient had no discomfort during the procedure, and postoperative neurological examination showed normal sensation and motor function of all four limbs (Figure 4).

#### Rationale for timing and indications of PKP

PKP was performed on postoperative day 11 following diaphragmatic repair. This timing was determined based on a balanced consideration of surgical safety and clinical necessity. First, the 11-day interval allowed sufficient recovery of respiratory function, removal of the chest drain, and initial healing of the diaphragmatic suture line, thereby minimizing the risk of excessive diaphragmatic tension or suture disruption caused by elevated intra-abdominal and intrathoracic pressure during prone positioning for spinal augmentation (25). Second, an initial trial of conservative management including strict bed rest and standardized analgesic therapy was administered during this period; however, persistent severe back pain [Visual Analogue Scale (VAS) score = 7] and markedly restricted mobility indicated failure of conservative treatment. Third, multidisciplinary evaluation confirmed that the patient's general condition was medically optimized and that she had adequately recovered from thoracic surgery to safely tolerate a second elective procedure. Finally, prolonged bed rest for osteoporotic vertebral fractures is associated with a significantly elevated risk of hypostatic pneumonia, venous thromboembolism, and other immobilization-related complications. Therefore, PKP was performed as soon as diaphragmatic healing was deemed reliable to facilitate early mobilization and reduce systemic complication risks (21).

The indication for PKP over continued conservative management was based on integrated clinical and radiological criteria: persistent disabling back pain refractory to standardized analgesia, 32.4% loss of T12 vertebral body height, MRI-confirmed acute vertebral bone marrow edema, and an intact posterior vertebral wall with low risk of intraspinal cement leakage. The patient's preference for early functional recovery was also incorporated into the decision-making. This treatment strategy was individualized to the patient's unique combined injury profile (26–28).

### Follow-up and outcomes

#### Thoracic outcomes

**Figure 5:**
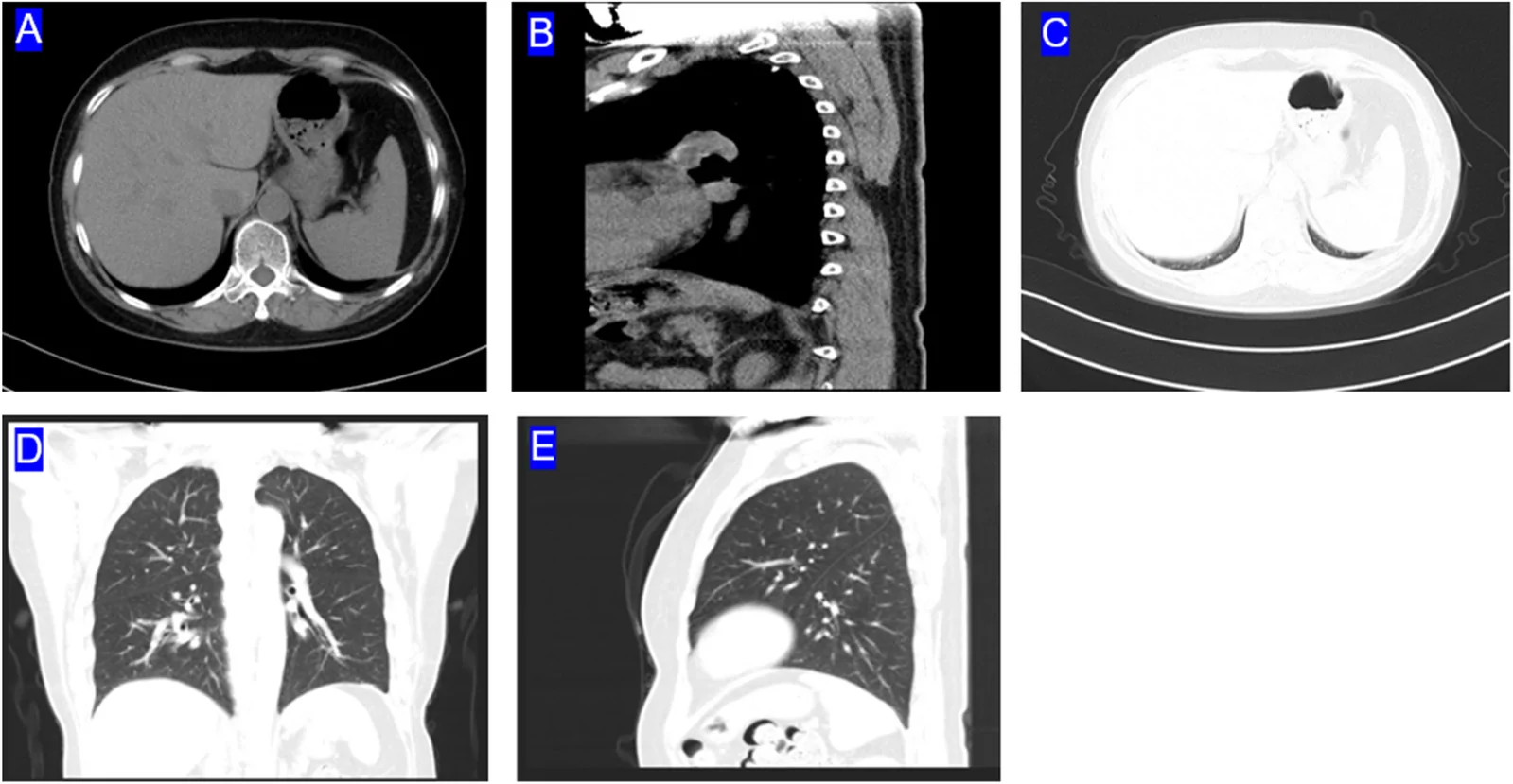
Three-month follow-up chest CT images **(A–E)** in sagittal, coronal, and axial views, demonstrating complete repair of the diaphragmatic hernia with no recurrence and full re-expansion of the left lung.

- Postoperative chest CT confirmed complete repair of the diaphragmatic defect and full re-expansion of the compressed left lower lobe.
- Abdominal discomfort resolved gradually, with no symptoms of bowel obstruction or respiratory distress.
- At 3-month follow-up, chest CT showed no recurrence of diaphragmatic hernia, with full expansion of the left lung (Figure 5).

#### Spinal outcomes

**Figure 6:**
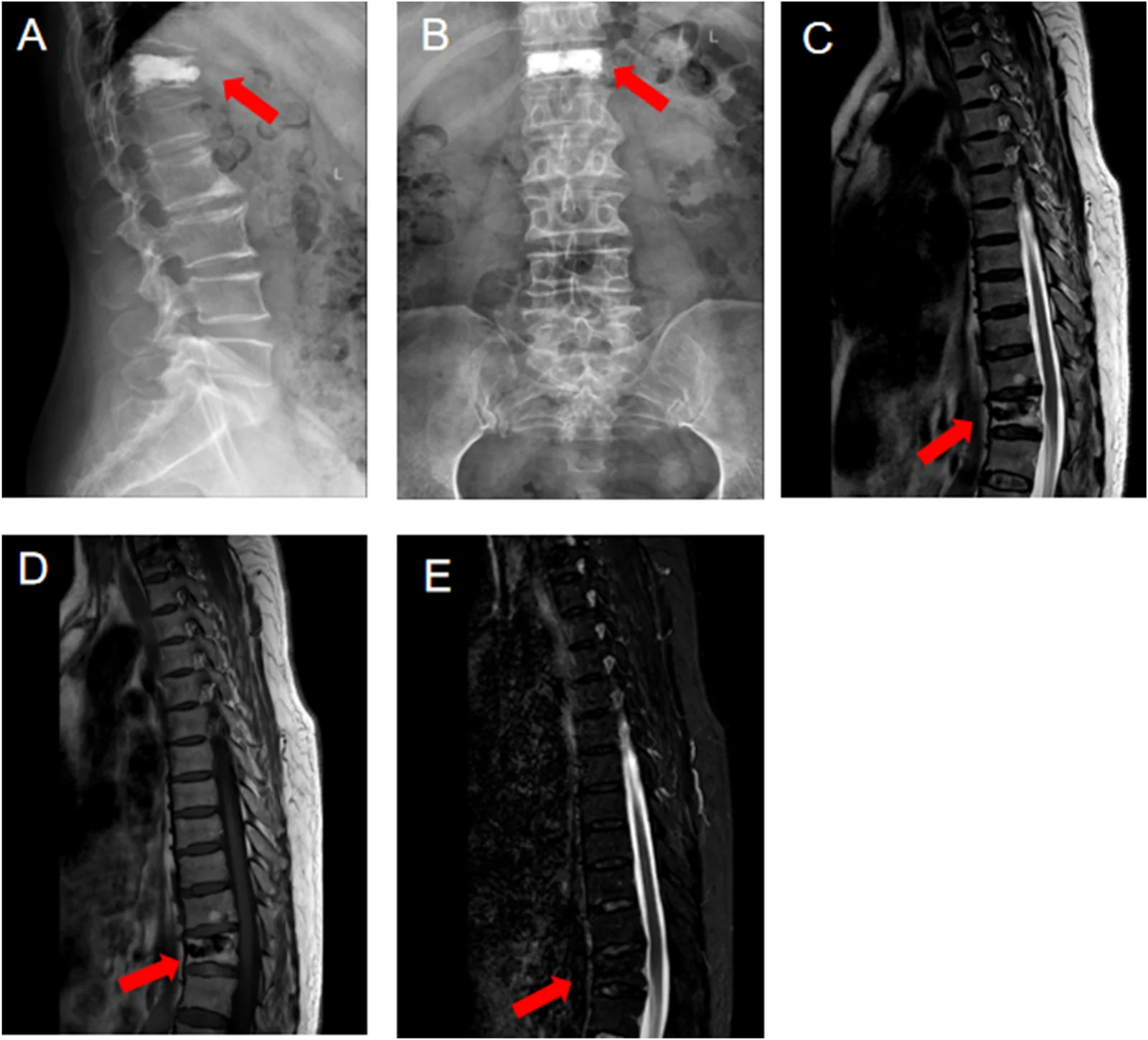
Three-month follow-up plain radiography and MRI images **(A–E)** demonstrating significant restoration of vertebral height and progressive bony healing of the T12 compression fracture.

- Back pain Visual Analogue Scale (VAS): 7 points before PKP; 4 points 1 day after PKP; 1 point at discharge; 0 points at 3-month follow-up.
- Oswestry Disability Index (ODI): 68 points (severe disability) at baseline; 22 points (moderate disability) at discharge; 8 points (minimal disability) at 3-month follow-up.
- Postoperative plain radiography showed satisfactory vertebral height restoration and good cement position.
- At 3-month follow-up, imaging showed progressive bony healing of the T12 fracture, with no cement-related complications (Figure 6).

## Patient perspective

During follow-up, the patient's back pain and abdominal discomfort resolved gradually, with satisfactory recovery of physical function. The patient reported satisfaction with the minimally invasive surgical trauma, intraoperative blood loss, pain management, and overall recovery speed. No hemostatic device-related adverse events were identified.

### Novelty of the case: comparison with published literature

As shown in the Table 2, most previously reported cases resulted from high-energy trauma and were managed with open surgery. The novelty of the present case lies in three aspects:

1. It occurred after low-energy ground-level fall in a patient with osteopenia, which is extremely rare in published literature.
2. Intraoperative findings support acute exacerbation of a pre-existing chronic diaphragmatic defect, rather than pure acute traumatic rupture.
3. Staged minimally invasive treatment (VATS for hernia repair followed by PKP for vertebral fracture) was applied, achieving satisfactory outcomes with minimal trauma.

**Table 2:** Summary of reported cases of traumatic diaphragmatic hernia with or without associated thoracolumbar vertebral fractures.

Study	Year	Mechanism of injury	Fracture level	Defect side	Herniated organs	Diagnosis timing	Treatment approach	Outcome
Rathnayaka et al. (7)	2021	Motorbike accident (high-energy)	T12–L1 fracture-dislocation with spinal cord injury	Left	Jejunum, spleen	Delayed (4 days post-injury)	Emergency laparotomy + diaphragmatic repair; delayed spinal fixation	Good recovery, ongoing rehabilitation
Kishore et al. (9)	2018	High-velocity blunt trauma	No vertebral fracture	Left	Stomach, small bowel, colon, omentum	Acute	Emergency laparotomy, primary suture repair (1-0 proline)	Good recovery, discharged within 1 week
Gielis et al. (18)	2022	Road traffic accident (car vs lamppost, 70 km/h)	No vertebral fracture	Left	Stomach	Acute	Laparoscopic repair with mesh reinforcement	Uneventful recovery, no recurrence at 12 months
Yadav et al. (29)	2023	Fall injury (6 months prior)	No vertebral fracture	Right	Ileum (gangrenous, with perforation)	Delayed (6 months, with obstruction and perforation)	Exploratory laparotomy, primary diaphragmatic repair, ileal resection, double-barrel ileostomy	Successful recovery, discharged after 28 days
Alfaraj et al. (30)	2023	Fall from height (construction roof)	No vertebral fracture (tibia/fibula open fracture)	Left	Abdominal organs with mesenteric tear	Acute	Exploratory laparotomy, diaphragmatic repair, mesenteric repair, external fixation	Deceased (day 10 in ICU, multi-organ failure)
Present case	2023	Ground-level fall (low-energy)	T12 compression fracture (osteopenia)	Left	Greater omentum, colon	Acute (<48 h after admission)	Staged minimally invasive: VATS repair → PKP on day 11	Excellent recovery, no recurrence at 3 months

## Discussion

A T12 vertebral compression fracture combined with diaphragmatic hernia is a rare clinical condition. When the body experiences vertical forces such as those from a fall, these forces are transmitted through the spine, resulting in compression fractures of the thoracolumbar vertebrae, with the T12 vertebra at the thoracolumbar junction being the most commonly affected (8). In the present case, the patient suffered a ground-level fall, which caused a T12 vertebral compression fracture and was associated with acute exacerbation of a left diaphragmatic defect. This finding suggests the possibility that patients with low-energy trauma-induced thoracolumbar fractures may experience acute exacerbations of pre-existing, asymptomatic chronic diaphragmatic defects (9). Based on the intraoperative finding of adhesions and the low-energy mechanism of injury, this case may represent acute exacerbation of a pre-existing or chronic diaphragmatic defect rather than a purely acute traumatic rupture (10).

Several intraoperative findings support the interpretation of a chronic defect with acute exacerbation rather than a purely acute traumatic rupture. First, the herniated omental and colonic tissues demonstrated localized adhesions to the diaphragmatic defect margins, which would not be expected in an acute traumatic tear. Second, the defect edges appeared fibrotic and chronic rather than fresh and sharp. Third, the patient sustained only a low-energy ground-level fall, which is less likely to cause acute diaphragmatic rupture in the absence of pre-existing weakness. Taken together, these findings suggest that the patient likely had an asymptomatic diaphragmatic defect or diaphragmatic weakness resulting from a previous unrecognized minor injury, through which omental tissues gradually herniated and formed adhesions over time (11, 12). The subsequent fall with transient elevation of intra-abdominal pressure may have further enlarged the pre-existing hernia or acutely exacerbated the diaphragmatic defect, resulting in abdominal symptoms and decreased left lung volume (11, 13).

The diagnosis of diaphragmatic pathology concurrent with vertebral fracture presents notable clinical challenges. Symptoms of diaphragmatic hernia are often masked by concurrent injuries, and previous studies have noted that a proportion of diaphragmatic injuries are not diagnosed at initial presentation (7). The initial clinical focus was appropriately directed toward the evident vertebral fracture. Several factors contributed to the relatively subtle clinical presentation, resulting in the failure to identify the diaphragmatic hernia immediately upon admission. In this case, the patient exhibited no classic diaphragmatic hernia symptoms upon admission, such as severe respiratory distress, nausea, vomiting, or gastrointestinal obstruction (14, 15). Furthermore, the diaphragm possesses a robust physiological compensatory capacity that further masks clinical symptoms; the chronic adhesions observed in this case may additionally stabilize the herniated organs and delay the onset of decompensation (20). Second, the patient's mild abdominal pain was likely caused by tractional stimulation of the pleura and peritoneum following diaphragmatic rupture. This nonspecific pain could easily be confused with pain from other soft tissue injuries after trauma (16). Third, the severe thoracolumbar pain caused by the vertebral fracture diverted clinical attention, further masking the mild, atypical symptoms of DH. Additionally, the left diaphragm is more prone to rupture due to the absence of protective liver coverage, consistent with the patient's left-sided DH (17).

With regard to diagnostic imaging, chest radiography remains the initial screening modality for diaphragmatic pathology; however, its sensitivity is limited, particularly in patients with small diaphragmatic defects without evident organ herniation. In this case, the chest radiograph demonstrated only an elevated left hemidiaphragm and reduced lung volume, which served as the critical clue prompting further evaluation. Chest CT is currently the gold standard for diagnosing diaphragmatic defects, clearly demonstrating diaphragmatic continuity, detecting minor defects, identifying herniated organs, and evaluating complications such as incarceration or strangulation (17). In this case, the chest x-ray indicated only an elevated left diaphragm, and the presence of traumatic diaphragmatic hernia (TDH) was confirmed through subsequent chest CT.

Regarding treatment, the cornerstone of TDH management is early surgical repair, as diaphragmatic defects do not heal spontaneously. Even minor diaphragmatic tears may progressively enlarge due to fluctuations in intra-abdominal pressure, potentially leading to serious complications such as incarceration, strangulation, and organ necrosis (18, 19). For patients with a combined T12 vertebral compression fracture and TDH, treatment priority should be determined based on the severity of each injury. In this case, despite the absence of typical TDH symptoms, a diaphragmatic defect existed, posing a potential risk of sudden herniation exacerbation and organ incarceration, which can be life-threatening. Therefore, thoracoscopic diaphragmatic hernia repair was prioritized, followed by PKP for vertebral fracture treatment after the patient's condition stabilized. This therapeutic strategy not only promptly mitigated the life-threatening risks associated with TDH but also ensured the safety of subsequent spinal surgery. Thoracoscopic minimally invasive surgery, characterized by clear surgical visualization, reduced trauma, minimal intraoperative blood loss, and rapid postoperative recovery, is the preferred surgical approach for TDH patients without significant intra-abdominal organ injury (5). PKP is a well-established minimally invasive surgical technique for compression fractures, effectively alleviating pain, restoring vertebral height, and improving spinal stability with proven efficacy and safety (1, 2). The patient's favorable recovery confirmed the safety and effectiveness of this sequential treatment strategy.

## Conclusion

T12 vertebral compression fracture combined with diaphragmatic hernia is a rare thoracoabdominal injury that is easily overlooked, especially in patients with atypical symptoms and low-energy trauma. Clinicians should maintain a high index of suspicion for combined diaphragmatic injury in patients with thoracolumbar compression fractures. Chest CT should be performed promptly when chest radiography shows abnormal findings or when patients present with nonspecific chest or abdominal symptoms, to achieve early diagnosis. For such patients, prioritizing diaphragmatic hernia repair to eliminate potential life-threatening risks, followed by staged minimally invasive treatment of vertebral fractures, is a safe and effective strategy that achieves favorable clinical outcomes.

## Data Availability

The original contributions presented in the study are included in the article/Supplementary Material, further inquiries can be directed to the corresponding authors.
